# The role of antifreeze genes in the tolerance of cold stress in the Nile tilapia (*Oreochromis niloticus*)

**DOI:** 10.1186/s12864-023-09569-x

**Published:** 2023-08-23

**Authors:** Abdel-Fattah M. El-Sayed, Asmaa A. Khaled, Amira M. Hamdan, Sara O. Makled, Elsayed E. Hafez, Ahmed A. Saleh

**Affiliations:** 1 Oceanography Department, Faculty of Science, Alexandria University, Alexandria City, Egypt; 2 Animal and Fish Production Department, Faculty of Agriculture (Saba Basha), Alexandria University, Alexandria City, 21531 Egypt; 3 Arid Lands Cultivation Research Institute, City of Scientific Research and Technological Applications, New Borg El Arab, Alexandria City, 21934 Egypt; 4 Animal and Fish Production Department, Faculty of Agriculture (Alshatby), Alexandria University, Alexandria City, 11865 Egypt

**Keywords:** Nile tilapia, Cold stress, Antifreeze protein, Cloning and sub-cloning, Differential Display-PCR

## Abstract

**Background:**

Tilapia is one of the most essential farmed fishes in the world. It is a tropical and subtropical freshwater fish well adapted to warm water but sensitive to cold weather. Extreme cold weather could cause severe stress and mass mortalities in tilapia. The present study was carried out to investigate the effects of cold stress on the up-regulation of antifreeze protein (AFP) genes in Nile tilapia (*Oreochromis niloticus*). Two treatment groups of fish were investigated (5 replicates of 15 fish for each group in fibreglass tanks/70 L each): 1) a control group; the fish were acclimated to lab conditions for two weeks and the water temperature was maintained at 25 °C during the whole experimental period with feeding on a commercial diet (30% crude protein). 2) Cold stress group; the same conditions as the control group except for the temperature. Initially, the temperature was decreased by one degree every 12 h. The fish started showing death symptoms when the water temperature reached 6–8 °C. In this stage the tissue (muscle) samples were taken from both groups. The immune response of fish exposed to cold stress was detected and characterized using Differential Display-PCR (DD-PCR).

**Results:**

The results indicated that nine different up-regulation genes were detected in the cold-stressed fish compared to the control group. These genes are Integrin-alpha-2 (*ITGA-2*), Gap junction gamma-1 protein-like (*GJC1*), WD repeat-containing protein 59 isoform X2 (WDRP59), NUAK family SNF1-like kinase, G-protein coupled receptor-176 (*GPR-176*), Actin cytoskeleton-regulatory complex protein pan1-like (*PAN-1*), Whirlin protein (*WHRN*), Suppressor of tumorigenicity 7 protein isoform X2 (*ST7P*) and ATP-binding cassette sub-family A member 1-like isoform X2 (*ABCA1*). The antifreeze gene type-II amplification using a specific PCR product of 600 bp, followed by cloning and sequencing analysis revealed that the identified gene is antifreeze type-II, with similarity ranging from 70 to 95%. The in-vitro transcribed gene induced an antifreeze protein with a molecular size of 22 kDa. The antifreeze gene, *ITGA-2* and the *WD* repeat protein belong to the lectin family (sugar–protein).

**Conclusions:**

In conclusion, under cold stress, Nile tilapia express many defence genes, an antifreeze gene consisting of one open reading frame of approximately 0.6 kbp.

**Supplementary Information:**

The online version contains supplementary material available at 10.1186/s12864-023-09569-x.

## Background

The Nile tilapia (*Oreochromis niloticus*) is one of the most important farmed fishes in the world, taking the 2^nd^ most cultivated fish in aquaculture after carp [1]. Global tilapia aquaculture has witnessed significant expansion during the past three decades [1, 2]. Currently, tilapia culture is practised in more than 130 countries worldwide, even in areas beyond their ecological tolerance [3]. As a result, the global production of farmed tilapia boosted from 383,654 tonnes in 1990 (2.28 percent of total aquaculture production) to 5,670,981 tonnes in 2015, representing 7.4% of global aquaculture (excluding aquatic plants) and 11.63% of total finfish aquaculture (FAO, 2017). Tilapia production is forecasted to grow 3.7% in 2022/2023, breaking the 6,000,000 metric tonnes barrier according to the Global-Seafood-Alliance (GSA, 2022).

Nile tilapia is a tropical and subtropical eurythermal freshwater fish, which can tolerate a wide range of environmental temperatures, ranging from a lower lethal of 7–10 °C to an upper lethal of 42 °C [4]. However, this fish is sensitive to low water temperatures. Long exposure of Nile tilapia to low temperatures (7–10 °C) affects the physiology, growth, reproduction and metabolism, especially in temperate and subtropical regions, which are characterized by seasonal fluctuations in water temperature [5–7]. Severe stress and mass mortalities have also been reported in tilapia due to severe cold weather [8, 9]. Tilapia feed intake is sharply reduced below 20 °C, and feeding stops at about 16 °C, while severe mortality occurs at 12 °C or lower [6, 10], leading to economic losses [7].

Cold stress may lead to the expression of ‘‘cold-induced genes’’ in teleost fishes. These genes contribute to the control and regulation of the acclimation responses in fish [11]. Several cold-responsive genes, including Antifreeze Proteins (*AFPs*) [12–14] have been isolated and characterized in different fish species.

Antifreeze proteins (*AFPs*) were identified in several organisms including animals, plants and insects. *AFPs* are biological substances found in polar fishes [14, 15]. They can control the shape and growth of ice crystals to cope with sub-zero temperatures in freeze-tolerant organisms [16, 17], and they have several main activities for them, such as; freezing point depression and ice recrystallization inhibition [18, 19]. Consequently, fish can inhabit ice-laden or cold water below the freezing points of their blood serum in presence of *AFPs* [20, 21]. Several types of *AFPs* (*I*, *II*, *III* and *IV*) have been discovered, classified and characterized [14]. The production of *AFPs* in cold-tolerant aquatic animals helps them to maintain body fluids in the liquid state at sub-zero temperatures [22]. Therefore, *AFP* genes may confer the property of cold tolerance to transgenic fishes. For example, transgenic salmon constructed by the introduction of *AFP-I* into eggs revealed freeze-resistant traits [23, 24]. Also, *AFP-III* microinjection exhibited significant cold resistance in goldfish [25], turbot fish (*Scophthalmus maximus*) [26] and mice [27].

Similarly, the administration of *AFPs* in temperate and tropical fish species may improve their cold resistance during cold seasons (e.g. during overwintering) [14, 28]. This assumption has been tested by Wu et al. [29] who studied the effect of *AFP* on cold tolerance in juvenile Mozambique tilapia (*Oreochromis mossambicus*) and milkfish (*Chanoschanos*) exposed to low temperatures. The fish were transferred from 26˚C to 13˚C. The mortality of *AFP*-injected fish was only 14% compared to 54% in the control group. So, *AFPs* could solve several of the cold problems [30]. These findings clearly demonstrate that *AFPs* can enhance the tolerance of tilapia to low temperatures [14, 28]. In addition, antifreeze proteins ‘‘Type I from winter flounder, Type-III from the ocean pout, and Antifreeze glycoprotein (AFGP) from Atlantic cod’’ increased the cold tolerance of goldfish exposed to various low temperatures [31, 32].

It has also been reported that cold shocks and cold acclimation may up-regulate several other genes including; *pak1ip1*, *per*2, *loc-100697261* and *loc-100696456* in zebrafish larvae, as means of adaptation to, and tolerance of, rapid decreases in environmental temperatures [33–35]. Also, Dunham et al. [36] found that the expression of several genes such as; *Hsp70*, *Hsp90*, *TB2*, *SG2NA*, and acyl-CoA binding protein in the brain of channel catfish, *Ictaluruspunctatus* were altered within 2 h following a decrease in water temperature from 24 to 12° C. Induced genes expressions suggest that multiple signal-transduction pathways are involved in cold acclimation. Likewise, Chen et al. [34] reported that cold shock-induced circadian gene expression in zebrafish since Period-circadian-protein-homolog-2* (per2)* was significantly regulator under cold shock. This finding suggests that circadian genes may serve as a protective way against cold stress.

However, it is not known whether cold stress would stimulate the expression of antifreeze protein genes in Nile tilapia (*Oreochromis niloticus*). Therefore, the present study was carried out in the Aquaculture facility, Oceanography Department, Faculty of Science, Alexandria University, Egypt to investigate the effects of cold stress on the up-regulation of *AFP* genes in juvenile Nile tilapia (*O. niloticus*).

## Results

### Fish acclimation to low water temperature

**Fig. 1:**
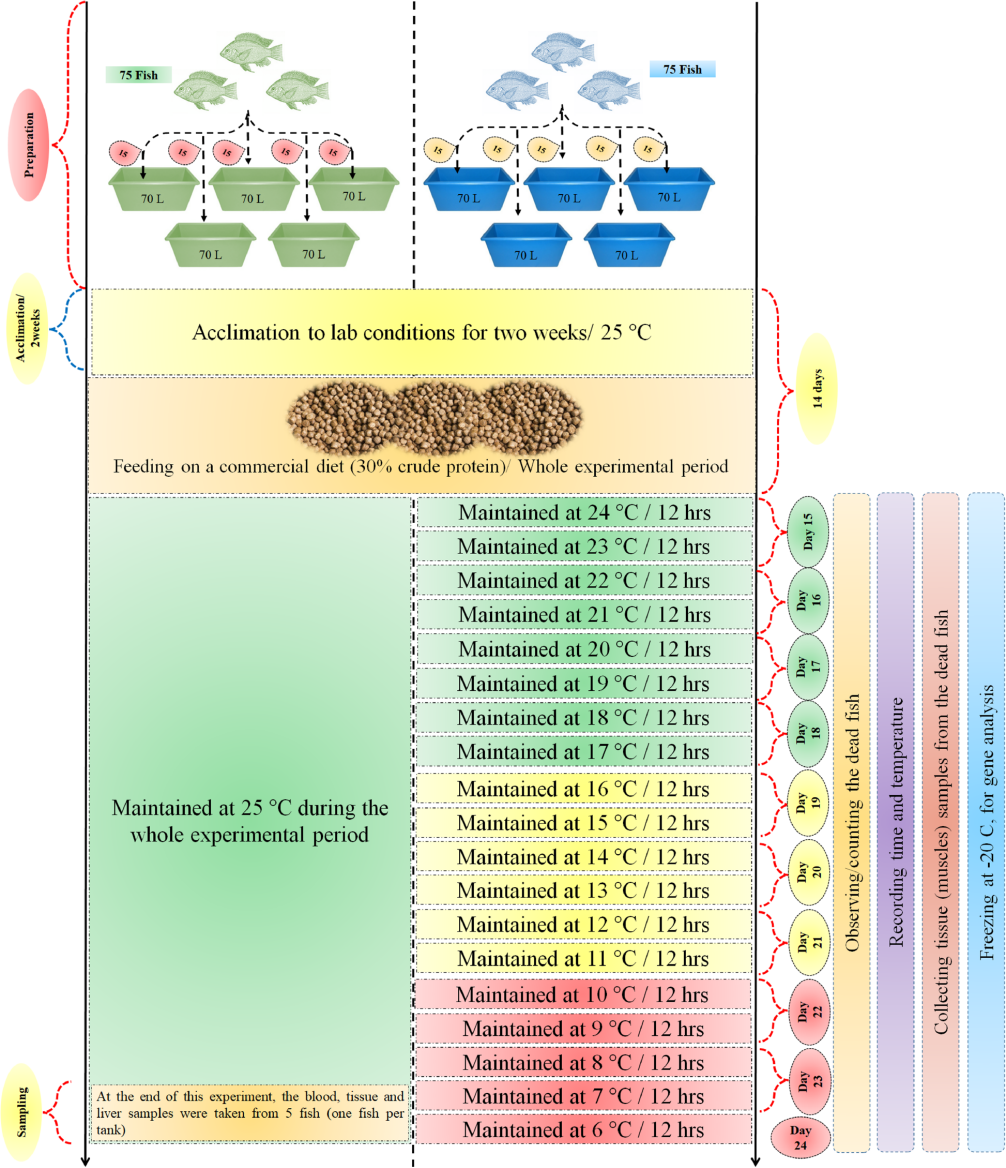
The experimental design

The experiment was designed professionally to obtain high accuracy results as shown in Fig. 1. In the current study, the behaviour and feeding activity of Nile tilapia were normal when the water temperature decreased from 25–20 °C the feed consumption and swimming activity started to decrease. At 15 °C the fish stopped eating and became slightly sluggish and the survivability rate was 80.00%. At 10 °C, the fish started losing orientation and swam very slowly near the bottom and the survivability rate was 22.66%. When the water temperature reached 8 °C the fish showed death symptoms, such as; losing direction, swimming on their backs, losing response and staying on the bottom on their sides, and the survivability rate was 13.33%. At 7 °C only one fish showed high resistance to extreme cold after 10 days of fish exposure to decreasing water temperature and the survivability rate was 1.33%, and the samples were collected from this fish to further Differential Display-PCR (DD-PCR) test. At 6 °C, all the fish died and the survivability rate was 0.00%. On the other side, the survivability rate was 93.33% in the control group (Table 1).

**Table 1:** The fish's behaviour responds to different conditions of temperatures

Groups	Tem	Behaviour	Survivability rate
**Control Group**	25 °C	Normal	100% at the beginning of the experiment 93.33% at the end of the Experiment
**Cold stress Group**	25 °C	Normal	100% at the beginning of the experiment
	20 °C	The feed consumption and swimming activity started to decrease	100.00%
	15 °C	The fish stopped eating and became slightly sluggish	80.00%
	10 °C	The fish started losing orientation and swam very slowly near the bottom and the survivability	22.66%
	8 °C	The fish showed death symptoms (losing direction, swimming on their backs, losing response, staying on the bottom on their sides)	13.33%
	7 °C	Only one fish showed high resistance to extreme cold after 10 days of fish exposure to decreasing water temperature	1.33%
	6 °C	All the fish died	0.00%

### Amplification, purification, manipulation and digestion

**Fig. 2:**
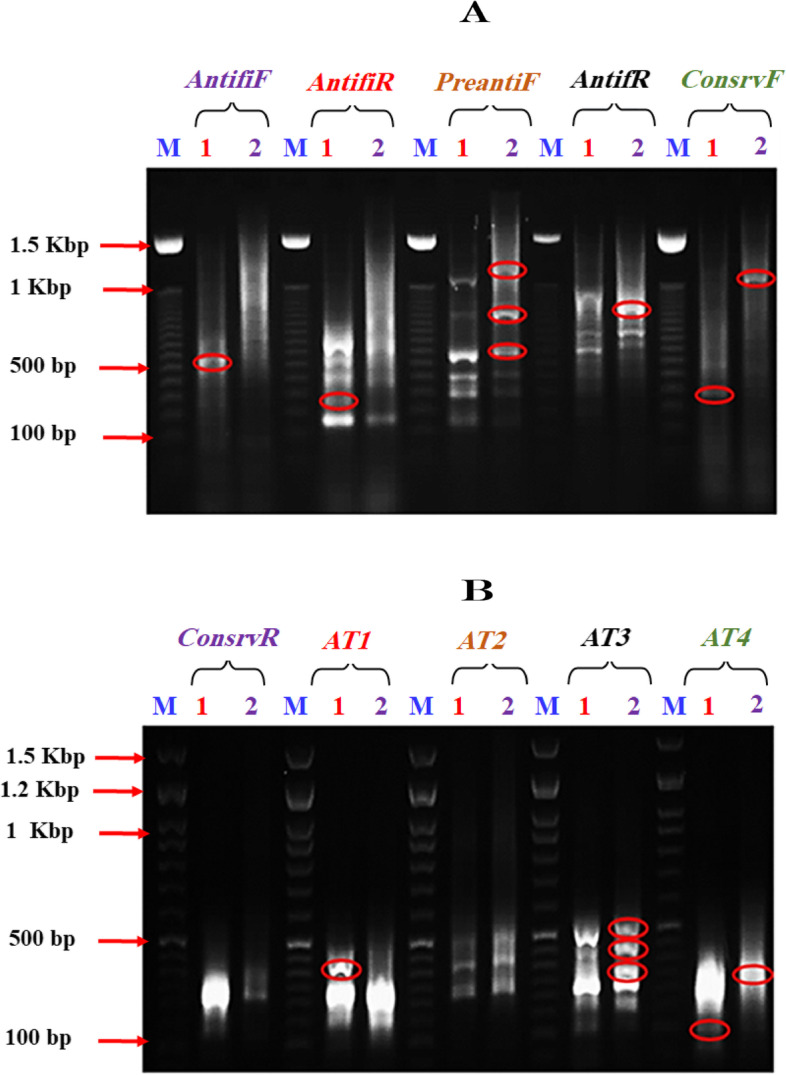
Differential display (DD-PCR) using 10 different primers as arbitrary primers; **A** (*AntifIF*, *AntifIR*, P*rentiF*, *AntifR* and *ConsrvF*). **B** (*ConsrvR*, *AT1*, *AT2*, *AT3* and *AT4*) for scanning the up and down regulated genes in the RNA of Nile tilapia (Oreochromis niloticus). Lanes; M: 1.5 Kbp DNA marker, Lane 1: Control group, Lane 2: Cold-stressed group

At the end of the cold-stress experiment, only one fish showed high resistance to extreme cold (at 7 °C) after 10 days of fish exposure to decreasing water temperature. The RNA of this fish was tested by the DD-PCR compared with the non-cold stressed fish. Data presented in Fig. 2; A and B revealed that there are two down-regulated bands (genes) in stressed fish when they were tested by the primers *Antifi*F and *AntifiR*. The molecular sizes of the two genes were 500 and 250 bp, respectively. However, in the case of primer *PreantiF* different up-regulated genes, with molecular sizes of 1100, 800 and 600 bp were recorded. Concerning the primer *PreantiR*, a band with a molecular size of 850 bp was up-regulated in the stressed fish, together with a down-regulated band with a molecular size of 200 bp. The primer *ConservR* did not succeed in amplifying more than 4 bands, one was upregulated with a molecular size of 300 bp, while the other with a molecular size of 200 bp was down-regulated. Additionally, no monomorphic bands were observed.

Four arbitrary primers (*AT1*, *AT2*, *AT3* and *AT4*) were used for studying the up and down-regulated genes (Fig. 2B). The results revealed that the band patterns obtained by these primers did not differ widely. Consequently, only one down-regulated gene was recorded with the primer *AT2* (400 bp), while three up-regulated genes were observed in the stressed fish tested by primer *AT4*.

**Fig. 3:**
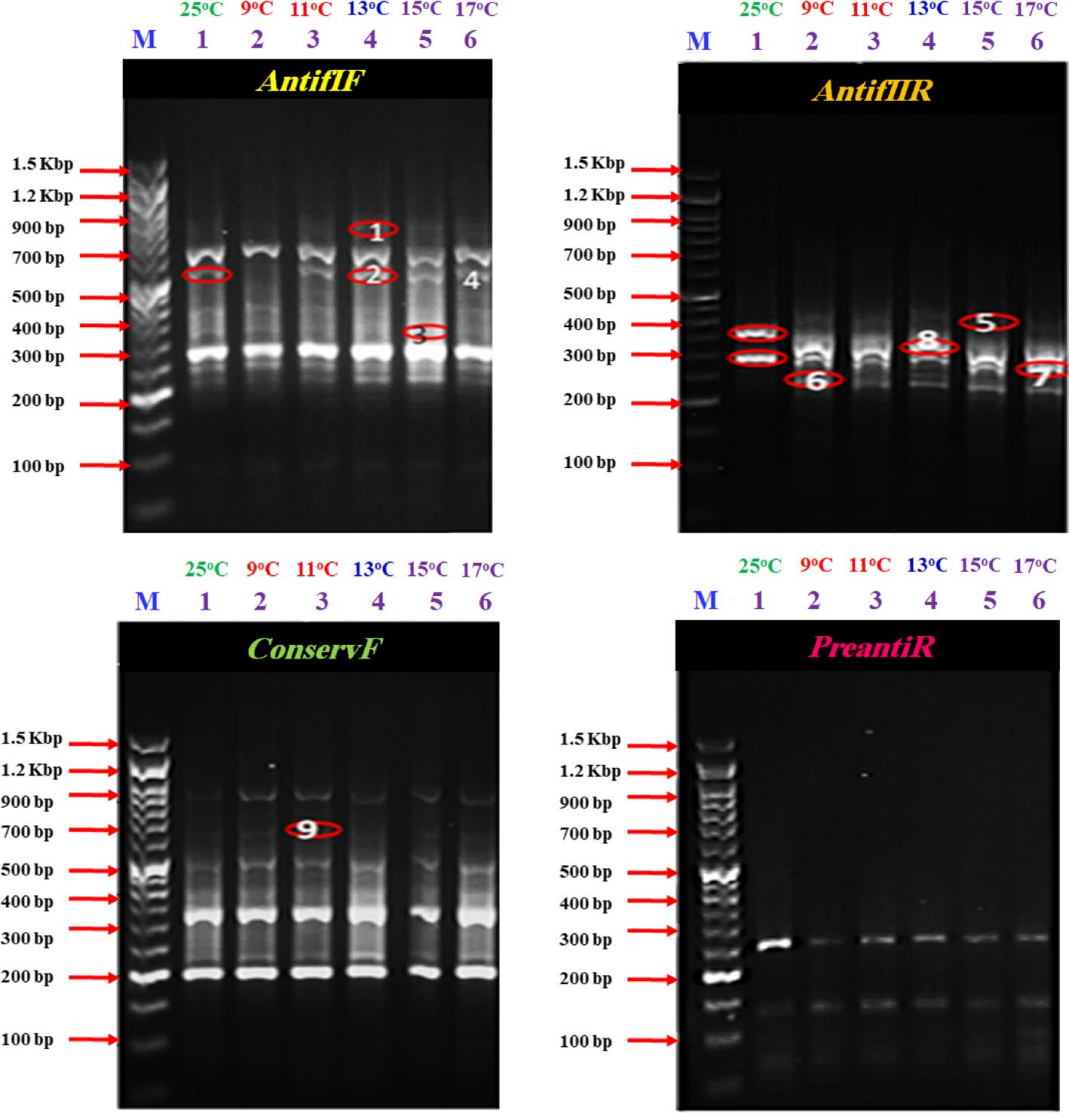
Differential display (DD-PCR) using four different primers (*Antif1F*, *AntifIIR*, *ConservF* and *PreantiR*) for scanning the up and down-regulated genes in the RNA of Nile tilapia exposed to different temperatures. M, 1.5 Kbp DNA Marker, Lane 1: Control; Lane 2: Fish exposed to 9 °C, Lane 3: Fish exposed to 11 °C, Lane 4: Fish exposed to 13 °C, Lane 5: Fish exposed to 15 °C and Lane 6: Fish exposed to 17 °C

According to the temperatures and behaviour of the fish (Table 1). A control fish sample at (25 °C), and five fish of the stressed group have been collected after dying at (9, 11, 13, 15 and 17 °C, respectively) and then compared together by amplifying four different arbitrary genes; *Antif1F*, *AntifIIR*, *ConservF* and *PreantiR* (Fig. 3 and S. File; Figs.; S1-S4). It was found that *AtifIF* primer succeeded to amplify 9 band patterns per lane. A band with a molecular size of 800 bp appeared in stressed fish sample no. 3 (lane 4/ at 13 °C); this band is an up-regulated one. A band with a molecular size of 600 bp appeared in both control and stressed fish sample no 3 (lane 4/ 13 °C), 4 (lane 5/ 15 °C) and 5 (lane 6/ 17 °C), but completely disappeared in stressed fish sample no.2 (lane 3/ 11 °C). On the other hand, the monomorphic band (250 bp) was presented in all examined fish. The expression of this band increased more than the control fish and was highest in the stressed fish sample no. 5.

Data obtained from primer *AtifIIR* showed different band patterns between the stressed and control fish samples. Two down-regulated genes (450 and 300 bp) were observed in the control group but disappeared in the stressed fish (450 and 300 bp). On the other side, two up-regulated genes (400 and 250 bp) were recorded in all the stressed, samples but were not presented in the control group. For primer *ConservF,* all the amplified bands were monomorphic except a band with a molecular size of 700 bp. Regarding the band patterns obtained by *PreantiR,* all the examined samples gave the same band patterns except for the control sample.

### DD-PCR Data analysis

**Fig. 4:**
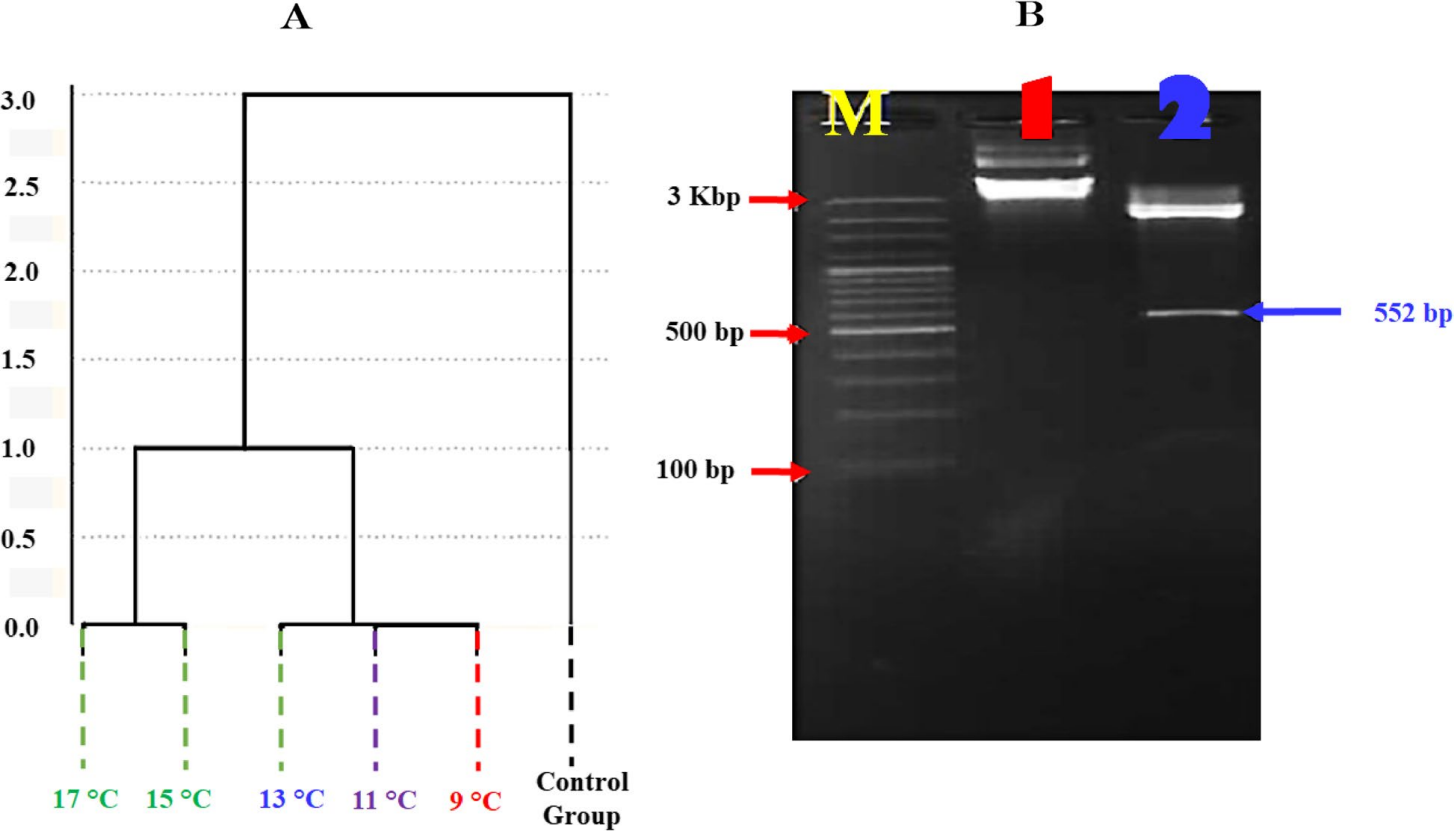
**A** Phylogenetic tree constructed based on the DD-PCR results for the cold-stressed Nile tilapia (*Oreochromis niloticus*) compared with the control group. **B** The recombinant antifreeze II gene. Lane 1: Plasmid DNA of *PUC57* recombinant vector contains the insert of the antifreeze gene. Lane 2: The released insert using *EcoRI* and *EcoRV* restriction enzymes

The present data were analysed based on the obtained band patterns of the five examined fish samples using four arbitrary primers. The results presented in Fig. 4A revealed that there is a genetic variation between the control (non-stressed) fish and the fish exposed to different temperatures. In this aspect, the phylogenetic analysis for DNA sequences was performed using C*lustalW* (1.82) *MEGA-6 V.4* software (http://en.bio-soft.net/tree/MEGA.html). The phylogeny was divided into two main clusters; the control sample formed one cluster, and the stressed fish formed the other. The degree of difference was 33% in the stressed fish compared to the control group. It was noticed that the variations among the three temperatures (9, 11 and 13 °C) were identical. The same observation was demonstrated between the two temperatures 15 and 17 °C.

### Sequence analysis of up-regulated genes

Based on the band patterns obtained by DD-PCR, some of the up-regulated genes (Fig. 3) were identified utilizing the used primers in the current study as; 1) Integrin-alpha-2 *(ITGA-2*) ‘antifreeze gene’. This gene is an up-regulated one, 2) Gap junction gamma-1 protein-like (*GJC1*), 3) WD repeat-containing protein 59 isoform X2 (*WDRP59*). The second and third genes are up-regulated ones, which are found in the cold-resistant genes presented in the genome of the cold-water fishes, 4) NUAK family SNF1-like kinase; which is well known as a stress-activated *kinase*, and is involved in tolerance of glucose starvation. 5) G-protein coupled receptor-176 (*GPR-176*). This gene is one of the immune response genes, which was induced in cold-stressed Nile tilapia to enable the fish to withstand cold stress. It is assumed that this gene is one of the transacting genes which help in opening the cold resistance genes. 6) Actin cytoskeleton-regulatory complex protein pan1-like (*PAN-1*) ‘plasma membrane gene’. These genes are responsible for releasing or receiving proteins through the plasma membrane. These proteins could be glycoproteins which acquire fish resistance against cold stress. 7) Whirlin protein (*WHRN*) ‘defensin gene’. This gene is one of the defence genes; induced to resist any stress, and is considered one of the innate immune systems. 8) Suppressor of tumorigenicity 7 protein isoform X2 (*ST7P*). This gene is an up-regulated one and its function has not yet been determined. 9) ATP-binding cassette sub-family A member 1-like isoform X2 (*ABCA1*). This gene helps in supporting the fish to resist cold stress.

### PCR amplification using a specific primer for the antifreeze gene (Type II)

**Fig. 5:**
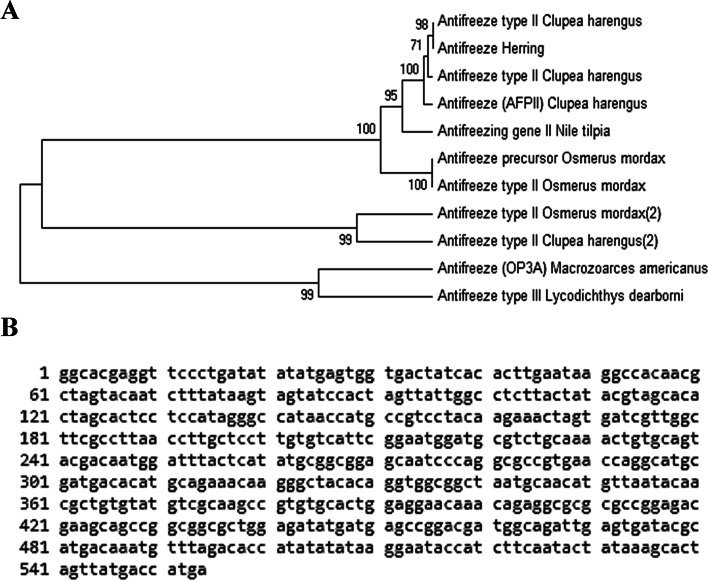
**A** Phylogenetic tree for the isolated antifreeze gene compared with the other antifreeze genes listed in the GenBank. The phylogeny was constructed using MEGA-6 V.4 software (http://en.bio-soft.net/tree/MEGA.html), UPGMA method. **B** A 550 bp sequence of *Anifreezing* gene from Nile tilapia (*NCBI accession no.*: OR269881)

The amplicon obtained by specific PCR using the antifreeze gene (Type II) was in a molecular size of 552 bp (Fig. 4B and S. File; Fig. S5). The PCR product was sequenced and the results revealed that the obtained sequence (AN: OR269881) is closely related to antifreeze genes (all types), with a similarity percentage ranging from 71 to 100%. However, the obtained DNA sequence showed high similarity with antifreeze gene type II (95 to 100%), (Fig. 5A).

### The phylogenetic tree between the antifreeze gene of Nile tilapia and other antifreeze genes listed in the *GenBank*

The obtained DNA sequence of the antifreeze gene of cold-stressed Nile tilapia in the current study (AN: OR269881) was aligned with 10 different antifreeze genes (belonging to the three categories of the antifreeze genes). It was found that the isolated gene is more closely related to the antifreeze gene isolated from herring (*Clupea harengus*), smelt (*Osmerus mordax*) and *Clupea harengus* (Fig. 5A). On the other hand, the two antifreeze genes isolated from Ocean pout (*Macrozoarces americanus*) and eelpout (*Lycodichthys dearbomi*) formed an outer group for the rest of the examined genes.

Another analysis was performed for both the isolated antifreeze gene II and the other up-regulated genes isolated by DD-PCR. The results indicated that *ITGA-2* and *WDRP59* are grouped with antifreeze type II isolated from Nile tilapia in the present study and another antifreeze type II of *C. harengus*, *O. mordix* and *Clupea harengus.* However, *ST7P* gene formed an outer group from this group. The following genes; *PAN-1*, *GPR-176* and *WHRN* are grouped with antifreeze gene II isolated from *O. mordax* and *C. harengus*. On the other hand, ATP-binding cassette protein was categorized with antifreeze gene III of *M. americanus* and *L. dearborni*.

### Determination of the recombinant antifreeze protein using SDS-PAGE

**Fig. 6:**
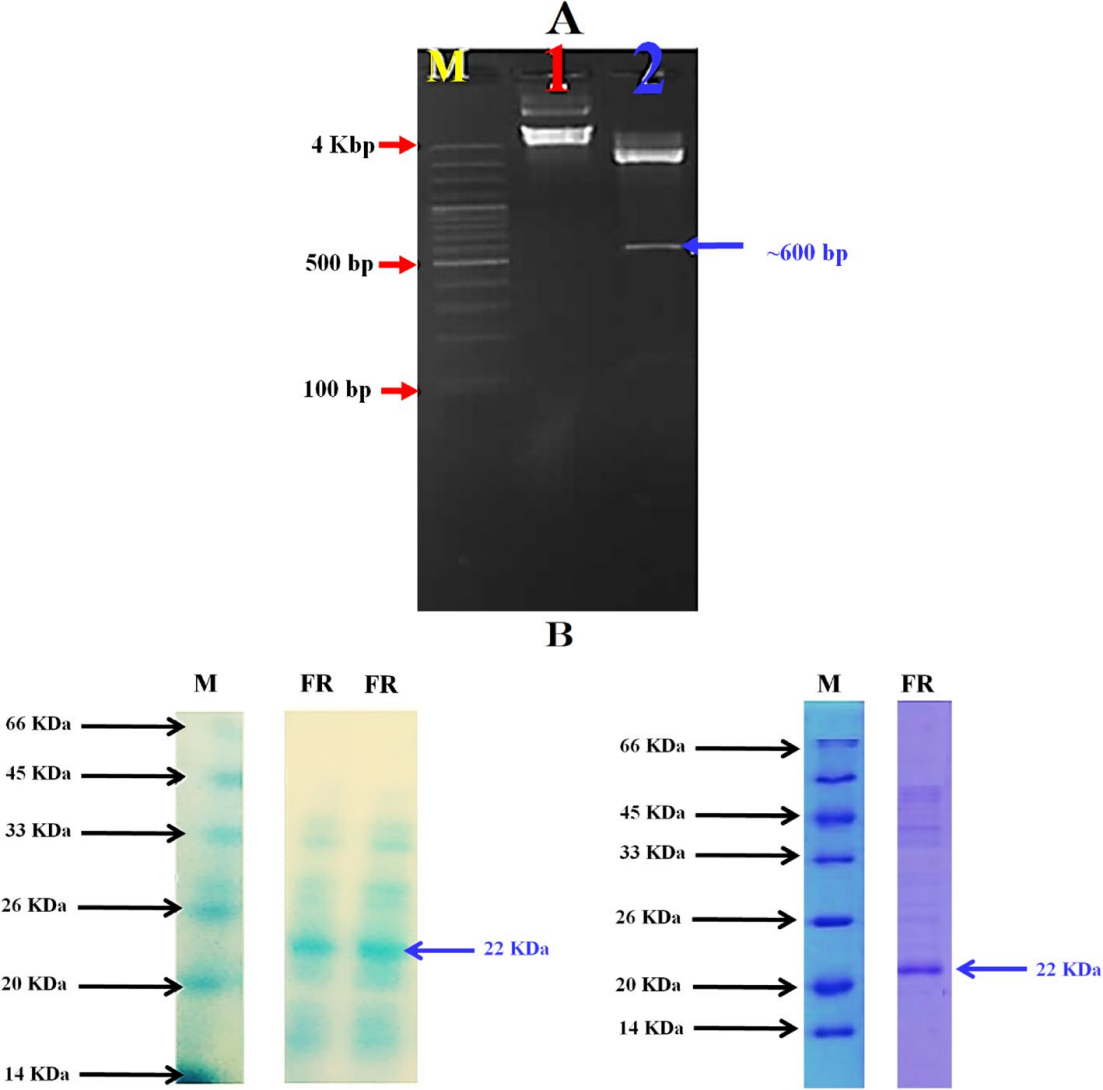
**A** Plasmid DNA of the cloned antifreeze gene in TOPO TA cloning vector. Lanes; M: 5Kbp DNA ladder, Lane1: TOPO TA cloning Vector contains the antifreeze gene, Lane 2: Double digestion of the recombinant DNA plasmid. **B** The recombinant protein of the antifreeze gene, Lane M: Low range protein Marker, Lanes 1 and 2: partial purified recombinant protein. Note: the full-length membranes and resolution for this figure can not be provided due to the modifications that have been done to the original figures

The recombinant *E. coli* cells containing the expression vector which harbours the antifreeze gene (Fig. 6A and S. File; Fig. S6) were induced using Isopentenyl transferase (*IPT*), and the resultant protein was purified (*See materials and methods section*). Protein was purified by Column Chromatogram and the purified protein was separated on 12% SDS-PAGE, as shown in Fig. 6B and S. File; Fig. S7, there is a band with a molecular size of 22 kDa.

**Fig. 7:**
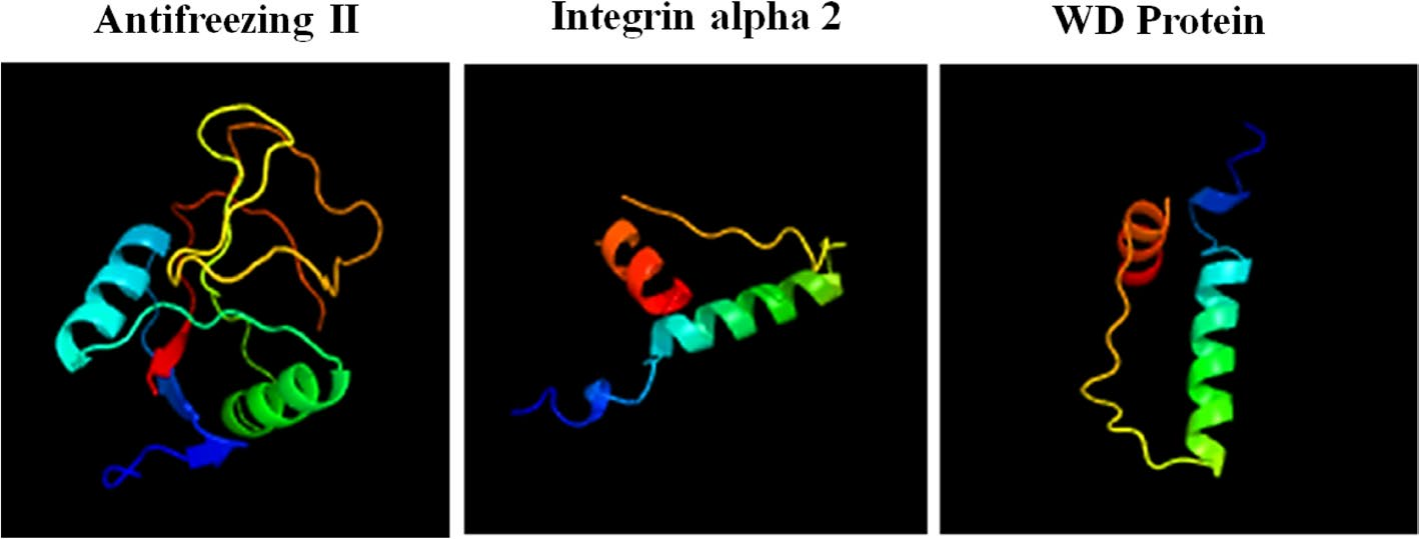
A 3D structure of the anti-freezing, alpha-integrin and WD proteins. The analysis was performed using Phyre2 (http://www.sbg.bio.ic.ac.uk/~phyre2/html)

The 3D structure of the anti-freezing, alpha-integrin and WD proteins is shown in Fig. 7. These findings were analysed and obtained with a help of the Phyre2 program. This type of analysis was carried out based on the obtained DNA sequence of studied anti-freezing genes. Data presented in Fig. 7 showed the 3D structure of the three suspected genes. The protein configuration of the three genes revealed their capabilities in developing the fish's immune response against abiotic stress, especially freezing. The anti-freezing gene, Integrin-alpha-2 and the WD repeat protein belong to the lectin family (sugar–protein) and lectins are essential components of innate immune response in animals, and can also regulate the adaptive immune responses in vertebrates as well.

## Discussion

Cold-shock stress in fish initiates a primary (neuroendocrine) response in the central nervous system (CNS), which stimulates the release of corticosteroid and catecholamine hormones [11]. Secondary responses include metabolic, cellular, osmoregulatory, haematological, and immunological changes [37, 38]. Cellular responses include the expression of heat shock proteins (HSPs) and *AFPs*. Tertiary responses refer to physiological and behavioural responses of the whole individual [35, 39].

The present study revealed that Nile tilapia subjected to cold stress (below 16 ºC) stopped eating at 10 ºC, the fish started losing orientation and swam very slowly near the bottom. At 7–8 ºC, death symptoms appeared, including swimming on their backs, losing response, staying on the bottom on their sides and then mortality occurred. Similar results were reported on goldfish exposed to stepwise reductions in temperature from 20 to 2 °C [40]. In addition, [41] reported that cold shock modulated catecholamine and cortisol concentrations and depressed phagocytic activity, leukocytes, antibody levels and plasma immunoglobulin M (IgM) in blue tilapia (*Oreochromis aureus*) subjected to a decrease in water temperatures from 25 to 12 °C.

Several molecular and physiological adaptations and changes occur to prepare the organism for environmental stresses, including signal transduction and activation of transcriptional factors that stimulate the synthesis of new proteins to cope with low temperatures [36, 42, 43]. Existing cellular proteins are also modified in response to the temperature shift through the synthesis of molecular chaperones [44]. Therefore, up-regulated genes in response to cold stress include antifreeze protein genes (*AFPs*) [45, 46].

Cold-stressed Nile tilapia in the present study expressed an antifreeze gene, and the obtained fragment showed high similarity to antifreeze genes (Type II). The expression of these genes suggests that cold-induced genes in Nile tilapia contribute to the control and regulation of the acclimation responses. The major categories of expressed genes include; 1) Antifreeze/cold-resistance genes (*ITGA-2*, *GJC1*, *WDRP59* and *ABCA1*), 2) stress-activated kinases (NUAK family SNF1-like kinase-2), 3) immune response genes (*GPR-176* and *WHRN*) and 4) cytoskeleton-regulatory (*PAN-1*).

The antifreeze gene, *ITGA-2* and the *WD* repeat protein belong to the lectin family (sugar–protein). Lectins refer to a group of sugar-binding proteins that can bind to the glycoproteins and glycolipids structures contained in biomembranes. This binding occurs independent of enzymes and immunoglobulins, and in turn agglutinates the cells to exert their biological function [47]. The lectins are essential components of innate immune response in animals, by inducing phagocytosis, activating platelet, initiating the complement system, and enhancing the natural killer cell activity. They can also regulate the adaptive immune responses in vertebrates [48–50].

On the other hand, the integrins are a large family of cell adhesion receptors, involved in muscle synthesis, freeze resistance [51] and immunological function [52, 53], such as early embryogenesis, regulation of cell proliferation and differentiation, leukocyte migration, and complement receptor-dependent phagocytosis [54]. The integrin-dependent effect on total signalling in a certain cell type can influence cytoskeletal arrangements, transcriptional activity and several metabolic reactions. Integrins can regulate the activity of paracrine loops of secreted growth factors [55]. They also influence a developmental process; however, this knowledge may not necessarily be tied to a specific integrin [56].

The expression of integrins representing in *ITGA-2* gene in the current study is evidence that Nile tilapia induce this gene in response to cold stress. Similarly, integrin genes have been reported in several fishes [57–59], for example, a beta 2 integrin molecule has been cloned from channel catfish [60]. A third complement protein component C3-opsonised particles have also been reported to increase the phagocytic activity of macrophages and neutrophils in salmon [61] and common carp [62]. Catfish neutrophils were also found to express a beta 2 integrin molecule [57]. These findings suggest that those receptors play a significant role in innate immune response in fish [52].

*The GJC1* gene is another cold-resistance gene, up-regulated in the present study. This gene is a member of the connexin gene family, composed of arrays of intercellular channels that provide a pathway for the diffusion of low molecular weight materials from cell to cell [63, 64]. They also regulate neuronal excitability, epithelial electrolyte transport and keratinocyte differentiation. These genes may, therefore, be directly related to ion and water balance [65], and thus, may play a significant role in fish osmoregulation. These types of genes might also permit the coordination of cellular events which favour the effective and timely response of the immune system [66]. Gaps are continuously synthesized and degraded, allowing tissues to rapidly adapt to changing environmental conditions. This may explain the up-regulation of Gap junction gamma-1 protein in Nile tilapia in the present study in response to decreasing water temperature.

Concerning the NUAK family SNF1-like kinase, it is a family of protein kinases that are activated by the LKB1. NUAKs have important roles in regulating cell adhesion, embryonic development, senescence, proliferation, neuronal polarity, axon branching [67, 68], gene transcription and resolution of the immune system [69]. NUAK is also well known as a stress-activated kinase. It plays an essential role in the regulation of cellular energy levels and is involved in tolerance to glucose starvation [70, 71].

It is well-documented that Nile tilapia stops feeding at temperatures lower than 16 °C. Therefore, prolonged exposure of these fish to cold stress in the present study led to starvation. Consequently, they required an indigenous source of energy to help in the induction of NUAK gene to compensate for the reduction of energy caused by starvation. In this regard, glycogen in the liver is an important source of glycerol production at low temperatures. In other words, low temperature depletes liver glycogen and activates glycerol accumulation through the initiation of glycerol antifreeze response [72]. The activation of glycogenolysis is associated with an increase in glycogen phosphatase (GPase) activity. In support, low temperature activated the initial glycerol antifreeze response in liver cells of rainbow smelt (*Osmerusmordax*) [72]. The fish accumulated high levels of glycerol in winter, which serves as an antifreeze.

As for G-protein coupled receptors, *GPR-176* plays an important role in the reproductive, nervous, endocrine, immune and cardiovascular systems, as well as its pathological roles in a diverse array of disorders [73]. Mostowy and Shenoy [74] reported that Actin cytoskeleton-regulatory complex protein pan1-like plays an important role as a defence gene in the plasma cell membrane. Similarly, *WHRN* may also stimulate the immune system response against either one or both ‘abiotic or biotic’ stresses [75].

WD-repeat-containing proteins form a very large family with diverse structures and functions. Functions of WD family include phygocytosis/actin binding, lymphocyte homing and cytoskeleton/myosin assembly. WD-repeat domains provide multiple protein–protein binding surfaces for reversible protein complex formation [76]. Also, *HAG* gene is a member of the family of F-box-WD-repeat proteins, in which the Fbox and WD-repeats are fused [77]. *HAG* gene has been cloned and characterized in East African cichlid fishes (*Labidochromis caeruleus*) [78]. This gene might function in the regulation of pigment-pattern formation. *WDRP59* has also been discovered in zebrafish larvae transcriptome when exposed to cold stress [79]. These findings supported the results in the present study, where *WDRP59* had up-regulation in Nile tilapia as a response to cold stress.

Various polar fish produce antifreeze proteins/peptides (*AFPs*) or antifreeze glycopeptides as a cold-adaptation mechanism for their protection against freezing damage [80]. Additionally, it was found that when seabream (*Sparus aurata*) embryos were injected with *AFP-I*, their cold resistance was significantly improved at 0 ºC. Also, antifreeze gene expression has been reported in other temperate and cold water fishes in response to cold stress [26]. These findings suggest that *AFPs* protect cellular structures by stabilizing cellular membranes.

Regarding DD-PCR results, reading the up and down-regulated genes observed in DD-PCR will help to understand how tilapia resist cold and how they are adapted to low-temperature stress. Generally, down-regulated genes indicate that metabolic activities are reduced when the fish are exposed to cold stress or after extended incubation of fish organs (brain) at low temperatures [36]. This means that fish respond to low temperatures by adjusting the expression of a large number of genes. So, extra investigations to examine *AFPs* in the Nile tilapia are needed to discover genetic variability locally and worldwide.

## Conclusion

In conclusion, Nile tilapia respond to cold stress by up-regulating the antifreeze gene with high similarity to type *II* antifreeze genes. The expression of this gene suggests that de novo synthesis of cold-induced proteins is necessary for the adaptation and tolerance of Nile tilapia to low water temperatures.

## Materials and methods

### Fish acclimation to low water temperature

Nile tilapia juveniles (20–25 g/fish) were obtained from a commercial fish farm (GPS; 31.042120, 30.853177). Two treatment groups of fish were investigated (5 replicates of 15 fish for each group): 1) the control group (*n* = 75); the fish were stocked in 5 fibreglass tanks (70 L each) and acclimated to lab conditions for two weeks and the water temperature was maintained at 25 °C during the whole experimental period with feeding on a commercial diet (30% crude protein). After the termination of the conditioning period, fish in each tank were counted and their physical appearance was checked to assure that they were healthy and do not have any injuries or deformities. As for the control group, at the end of the experiment, the tissues (muscles) samples were taken from 5 fish (one fish per tank). 2) Cold stress group; the same conditions as the control group except for the temperature, after the acclimation period. Initially, the temperature was decreased by one degree every 12 h (Fig. 1). At 16 °C the fish stopped eating, and therefore, feeding was stopped. Then, the water temperature was decreased by one degree every two days. When the water temperature reached 6–8 °C, the fish started showing death symptoms. In this stage the tissue (muscle) samples were taken from the cold stress group. Every death was observed, the time and the temperature degree were recorded then fish were removed from the cooling tanks, and the tissue samples were collected from the dead fish and frozen, for gene analyses. All experiments were performed under the supervision of the Animal Care and Use Committee of Alexandria University, and all efforts were made to minimize suffering. The methodology for collecting samples was as follows; a) the fish surface was sterilized with Clorox (5%) and then washed with sterile water three times. b) The sterilized fishes were opened using a sterilized scalpel under aseptic conditions and 1 mg of muscle tissues were transferred into a sterile Eppendorf tube (1.5 ml), and **c)** the tubes were labelled and stored under -20 until used further work.

### Primer designing, RNA and DNA extraction, amplification, purification and manipulation

#### Arbitrary and specific primers used in this study

Primers used in this study were listed in Table 2; where A1-A4 are arbitrary primers, while the other four primers are specific for several antifreeze genes. These primers were designed using the Primer 3 plus program (http://bioinfo.ut.ee/primer3-0.4.0) based on the *GenBank* data ‘‘Genome assembly O_niloticus_UMD_NMBU’’ (https://www.ncbi.nlm.nih.gov/datasets/genome/GCF_001858045.2). All of the listed primers in Table 2 were used to amplify the specific genes. The primers sequences and names were listed as; Antifreeze *IIF* and the expected amplicon is 934 bp. Another primer was designed based on the conserved regions among all the antifreeze genes type II isolated from cold-acclimated fish, and the amplicon had a molecular size of 450 bp (*Consrv)*. For the antifreeze gene type III, a set of primers was designed, where the expected amplicon was in a molecular size of 1408 bp (*Antifi)*. Concerning the pre-antifreeze gene, primers were designed to isolate amplicon with a molecular size of 3007 bp (*Preanti).* The specific primers were used as arbitrary primers in the differential display to examine the variations between the examined fish samples.

**Table 2:** The listing primer and sequence (5’ → 3’) that were used in the present study

Primers	Sequence 5` -`3	Annealing Temp	*Ref*
**At1**	ATTTCCTTGAAGAGAACGGTGC	53 °C	**GCF_001858045.2**
**At2**	AGTTCGGCCAGCATCTGCTCGT	35 °C	
**At3**	IIIIICGICGICATCTGGC	30 °C	
**At4**	ICGICTTATCIGGCCTAC	30 °C	
***ConsrvF***	ATATTGATCAAGGGTGGGGTGC	35 °C	
***ConsrvR***	CCTCCGCCTTCATCATCACTA	35 °C	
***AntifiF***	TCCCCAAACTAGTGGGAATGC	35 °C	
***AntifiR***	CCTTTGGGCTGACTTCCTCC	35 °C	
***PreantiF***	GCTCCAAATAACATCCTCCCT	30 °C	
***PreantiR***	TAAAGGTCCATAGGGGCATCTC	30 °C	
***Antifreezing IIF***	CTCTGTTCCAAACGCACCGA	55 °C	
***Antifreezing IIR***	ACCCAAATCACCCAAATCACAAC		

*II* Antifreeze protein Type 2

#### Differential Display PCR (DD-PCR)

DD-PCR has been widely used in biological research to investigate host–pathogen (Bio-stressed) interactions that can be demonstrated by studying gene regulation in stressed organisms [81]. DD-PCR in the present study was performed in two steps. The first step was on only one cold-stressed Nile tilapia compared with a control (non-stressed) fish. The second step was built up based on the data, where the antifreeze primers used as arbitrary primers could be used in DD-PCR and could scan more numbers of RNA than the other AT primers. DD-PCR was used in this study to examine the immune response of stressed fish compared with the control group.

#### RNA from tilapia tissues

Genomic RNAs were extracted from all Nile tilapia tissue (muscle) samples with RNeasy Mini Kit (QIAGEN, Germany). The resultant RNA was dissolved in diethylpyrocarbonate-treated water.

#### cDNA synthesis of extracted RNA

Reverse transcription reactions were performed in a reaction volume of 25 µl. The reaction mixture contained 2.5 µl of 5 × buffer with MgCl_2_, 2.5 µl of 2.5 mM dNTPs, 4 µl from oligo (dT) primer (20 p.ml/µl), 2 µg RNA and 200 U reverse transcriptase enzyme (MLV, Fermentas, USA). RT-PCR amplification was performed in a thermal cycler (Eppendorf, Germany) programmed at 42 °C for 1 h and 72 °C for 10 min and the cDNA was then stored at -20 °C until used.

#### DD-PCR Reaction constituents and conditions

Since cold stress in the current study was physical, the gene regulation in both stressed and non-stressed fish was examined using 10 arbitrary primers in DD-PCR (Table 2). The DD-PCR reaction was performed in a final volume of 25 µl of PCR reaction which consists of 2 µl of cDNA, 8.3 µl of Sterile Milli Q water, 2.5 µl of 5 × PCR reaction less buffer, 2.5 µl of 50 mM-MgCl_2_, 2.5 µl of 25 mM-dNTPs, 7 µl (10 pmol/µl) of each primer and 0.2 (5U/µl) *Taq* polymerase. PCR amplification was performed in a thermal cycler (Eppendorf, Germany) programmed for one cycle at 95 °C for 5 min, then 34 cycles as follows: 30 s at 95 °C for denaturation, one min at 35–40 °C for annealing and one min at 72 °C for elongation. Then the reaction was incubated at 72 °C for 10 min for the final extension. Two μl of loading dye were added prior to loading of 10 μl per gel slot. Electrophoresis was performed at 80 Volt with 0.5 × TBE as running buffer in 1.5% agarose/ 0.5 × TBE gels and then the gel was stained in 0.5 μg/ ml (w/v) Ethidium bromide solution and destained in deionized water. Finally, the gel was visualized and photographed using a gel documentation system (Alpha-chem. Imager, USA). The unique bands which appeared only in inoculated hosts (but not in the control group) were cut from the agarose gel and kept at -20 °C for further investigation.

#### DD-PCR data analysis

Pairwise genetic comparisons based on DD-PCR band patterns were made using Jaccard's similarity coefficient embedded with a help of NTSYS-pc Software [82, 83]. The polymorphic bands were scored as; 1 for the presence and 0 for the absence of the band. It was assumed that the bands with the same size were identical (monomorphic). Cluster analysis of the data matrix was performed by the Unweighted Pair Group Method with Arithmetic Means (UPGMA) with Jaccard’s similarity coefficient [84].

#### Isolation, purification, cloning and sequencing of several up-regulated genes

The PCR-amplified fragments were excised from the agarose gel and purified using a Qiagen Gel Extraction Kit (Qiagen, Germany). Purified DNA fragments were then cloned into PCR 4-TOPO vector (TOPO TA cloning kit, Invitrogen, USA) in competent *Escherichia coli (E. coli)* strain TOP 10. Plasmid DNA was isolated using a QIA-Spin mini-prep Kit (Qiagen, Germany). The sequence was performed on the recombinant DNA using the plasmid universal primers by BigDye Sequencing Kit and ABI 377 DNA sequencer (Colours Lab, Sigma, Egypt).

#### PCR amplification of the antifreeze gene using primers (Type II)

Based on DD-PCR results, the primers of the *Antifreezing II* gene gave better results than the other primers. Therefore, the genetic pool for some of the stressed fish was subjected to RNA extraction and cDNA synthesis, as mentioned previously. The synthesized cDNA of stressed fish was subjected to PCR amplification using *Antifreezing II* (Table 2). The 25 µl PCR reaction volume consists of 2 µl cDNA, 8.3 µl Sterile Milli Q water, 2.5 µl of 5 × PCR reaction less buffer, 2.5 µl of 50 mM-MgCl_2_, 2.5 µl of 25 mM-dNTPs, 7 µl (10 pmol/µl) of each primer, in addition to 0.2 (5U/µl) *Taq* polymerase. The PCR reaction conditions were denaturation at 95 °C for 2 min, followed by 34 cycles at 95 °C for 1 min, 550 °C for 1 min and 72 °C for 1 min. and final extension at 72 °C for 10 min. Amplified products were visualized in 1.5% agarose gel and run in 0.5 × TBE buffer.

#### Sequencing, alignments and phylogenetic analysis

Nucleotide sequencing of the antifreeze gene was done and submitted to the NCBI GenBank (AN: OR269881). Pairwise and multiple DNA sequence alignments were carried out utilizing *ClustalW* (1.82). Bootstrap neighbour-joining tree was generated using *MEGA-6 V.4*.

#### Cloning and sub-cloning of antifreeze gene (II)

The purified PCR product (550 bp) was cloned into the PUC57 cloning vector (GenScript, USA) using the double digestion of *Eco*RI and *Eco*RV restriction enzymes. Cloning and sub-cloning were performed according to Abdel-Fattah et al. [85].

#### Recombinant antifreeze protein extraction and purification

After 24 h of incubation, the induced recombinant bacteria cells were harvested by centrifuge at 12,500 rpm for 10 min at 4 °C. The cells were flooded with 2 ml of phosphate buffer (buffer Q, pH 7.5) and suspended cells were collected in a centrifuge tube. The cell suspension was sonicated on ice for 1 min and then centrifuged at 10,000 rpm for 30 min, and the supernatant was removed with a Pasteur pipette and purified by Column Chromatography (Sephadix G-50). The cells' free extraction was concentrated by ammonium sulfate precipitation before the purification process. The cells-free extract was precipitated by slow addition of solid ammonium sulfate (70% saturation) with constant stirring on a magnetic stirrer. All subsequent steps were carried out at 4 ºC for 30 min. The precipitate was separated by centrifugation at 13,000 rpm for 30 min at 4 °C, and the precipitated protein was re-suspended in 10 ml of 0.1 M sodium phosphate buffer (pH 6.8), and then dialyzed against excess of the same buffer.

#### Sephadex G-100 Gel Filtration Chromatography

The protein pellets obtained after saturation with ammonium sulphate (70%) were dissolved in gel-permeation buffer and filtered by 0.45 Millipore filter before loading into Sephadex G-50 column (2.4 × 75 cm) equilibrated with gel-permeation buffer. The flow rate was 1 m/min, and 2 ml fractions were collected continuously after zero time was reached. The contents of positive tubes corresponding to a single peak were pooled.

#### SDS-PAGE for the recombinant protein

To determine the purity of the obtained urease enzymes, Sodium Dodecyl Sulfate–Polyacrylamide Gel Electrophoresis (SDS-PAGE) was performed according to Sambrook et al. [86]. Samples buffer was added to each protein sample (35 μl) obtained from each purification step. Sample and a standard molecular weight marker (19—66 kDa, Sigma) were loaded on 12% SDS-PAGE. After denaturation at 95 °C for 5 min and electrophoresed at 100 V for 120 min, the gel was stained with Coomassie Brilliant Blue R-250 and destained overnight at room temperature in a destained solution with shaking. The gels were visualized and photographed using gel documentation system (Alpha-chem. Imager, USA).

### Supplementary Information

**Additional file 1:** **Figure S1.** Differential display (DD-PCR) using *Antif1F *primer for scanning the up and down-regulated genes in the RNA of Nile tilapia exposed to different temperatures. M, 1.5 Kbp DNA Marker. **Figure S2. **Differential display (DD-PCR) using *AntifIIR *primer for scanning the up and down-regulated genes in the RNA of Nile tilapia exposed to different temperatures. M, 1.5 Kbp DNA Marker. **Figure S3. **Differential display (DD-PCR) using *ConservF *primer for scanning the up and down-regulated genes in the RNA of Nile tilapia exposed to different temperatures. M, 1.5 Kbp DNA Marker. **Figure S4. **Differential display (DD-PCR) using *PreantiR *primer for scanning the up and down-regulated genes in the RNA of Nile tilapia exposed to different temperatures. M, 1.5 Kbp DNA Marker. **Figure S5. **The recombinant antifreeze II gene. Lane 1: Plasmid DNA of the PUC57 recombinant vector contains the insert of the antifreeze gene. Lane 2: The released insert using *Eco*RI and *Eco*RV restriction enzymes. **Figure S6. **Plasmid DNA of the cloned antifreeze gene in TOPO TA cloning vector. Lanes; M: 5Kbp DNA ladder, Lane1: TOPO TA cloning Vector contains the antifreeze gene, Lane 2: Double digestion of the recombinant DNA plasmid. **Figure S7. **The recombinant protein of the antifreeze gene, Lane M: Low range protein Marker, Lane FR: partial purified recombinant protein. Note: The full-length membranes and resolution for this figure can not be provided due to the modifications that have been done to the original figures.

## Data Availability

The datasets generated and/or analysed during the current study are available in the [Genbank] repository, with accession number [OR269881]”. Otherwise, the other related information/results are available in this manuscript and its supplementary information files.
