# Supplementary material for: The role of antifreeze genes in the tolerance of cold stress in the Nile tilapia (Oreochromis niloticus)
Source: BMC Genomics. 2023 Aug 23;24:476. doi: 10.1186/s12864-023-09569-x (PMC10464439; doi:10.1186/s12864-023-09569-x)
Supplement: Supplementary file 1 — Additional file 1: Figure S1. Differential display (DD-PCR) using Antif1F primer for scanning the up and down-regulated genes in the RNA of Nile tilapia exposed to different temperatures. M, 1.5 Kbp DNA Marker. Figure S2. Differential display (DD-PCR) using AntifIIR primer for scanning the up and down-regulated genes in the RNA of Nile tilapia exposed to different temperatures. M, 1.5 Kbp DNA Marker. Figure S3. Differential display (DD-PCR) using ConservF primer for scanning the up and down-regulated genes in the RNA of Nile tilapia exposed to different temperatures. M, 1.5 Kbp DNA Marker. Figure S4. Differential display (DD-PCR) using PreantiR primer for scanning the up and down-regulated genes in the RNA of Nile tilapia exposed to different temperatures. M, 1.5 Kbp DNA Marker. Figure S5. The recombinant antifreeze II gene. Lane 1: Plasmid DNA of the PUC57 recombinant vector contains the insert of the antifreeze gene. Lane 2: The released insert using EcoRI and EcoRV restriction enzymes. Figure S6. Plasmid DNA of the cloned antifreeze gene in TOPO TA cloning vector. Lanes; M: 5Kbp DNA ladder, Lane1: TOPO TA cloning Vector contains the antifreeze gene, Lane 2: Double digestion of the recombinant DNA plasmid. Figure S7. The recombinant protein of the antifreeze gene, Lane M: Low range protein Marker, Lane FR: partial purified recombinant protein. Note: The full-length membranes and resolution for this figure can not be provided due to the modifications that have been done to the original figures. [file 12864_2023_9569_MOESM1_ESM.pdf]

Supplementary File (1)

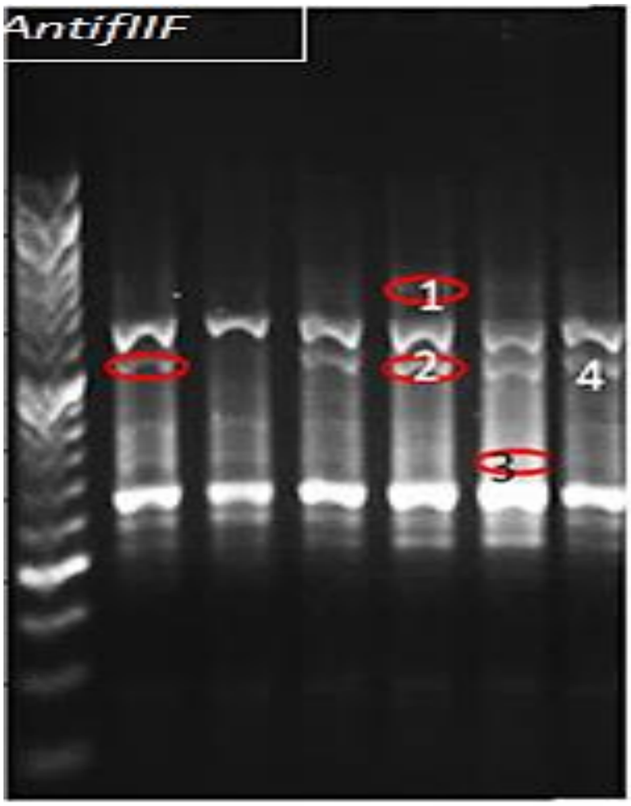

Figure S1. Differential display (DD-PCR) using *Antif1F* primer for scanning the up and down-regulated genes in the RNA of Nile tilapia exposed to different temperatures. M, 1.5 Kbp DNA Marker.

Supplementary File (1)

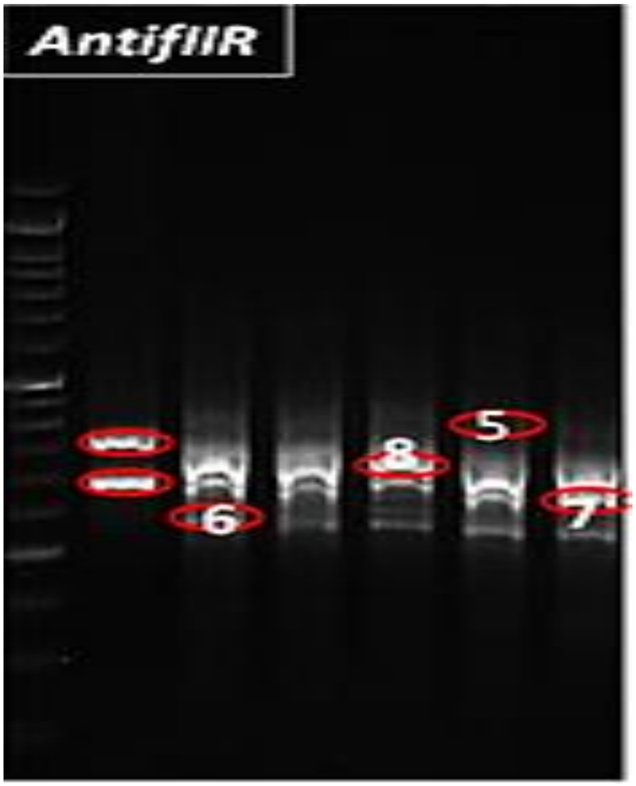

Figure S2. Differential display (DD-PCR) using *AntifIIR* primer for scanning the up and down-regulated genes in the RNA of Nile tilapia exposed to different temperatures. M, 1.5 Kbp DNA Marker.

Supplementary File (1)

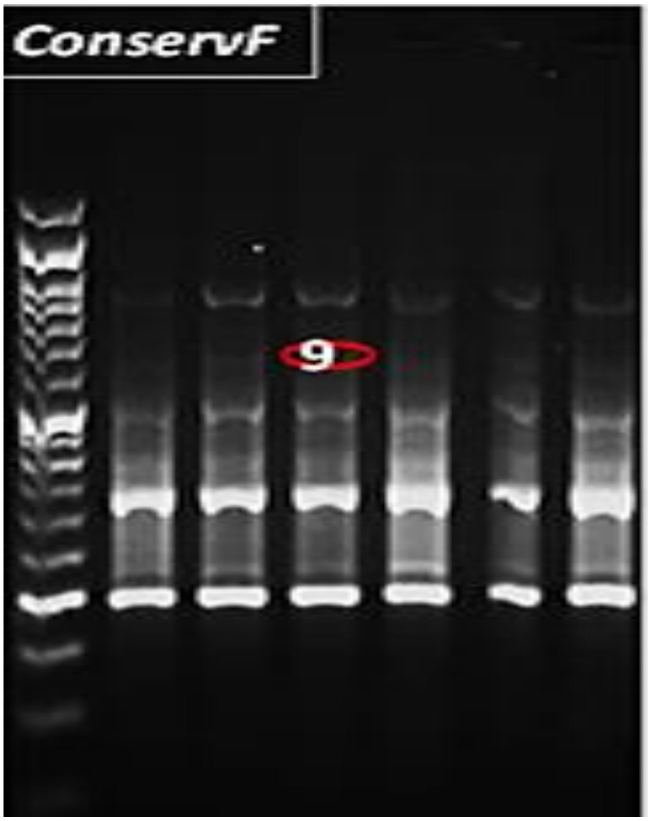

Figure S3. Differential display (DD-PCR) using *ConservF* primer for scanning the up and down-regulated genes in the RNA of Nile tilapia exposed to different temperatures. M, 1.5 Kbp DNA Marker.

Supplementary File (1)

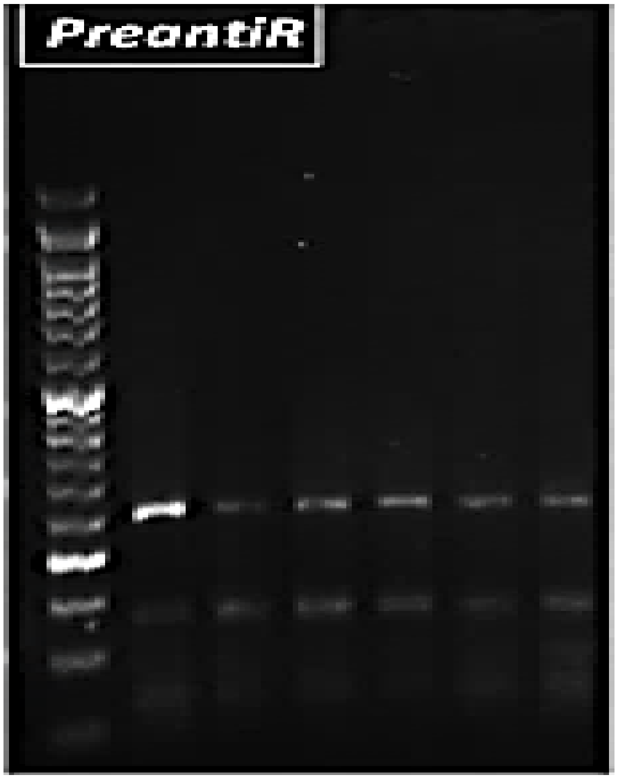

Figure S4. Differential display (DD-PCR) using *PreantiR* primer for scanning the up and down-regulated genes in the RNA of Nile tilapia exposed to different temperatures. M, 1.5 Kbp DNA Marker.

Supplementary File (1)

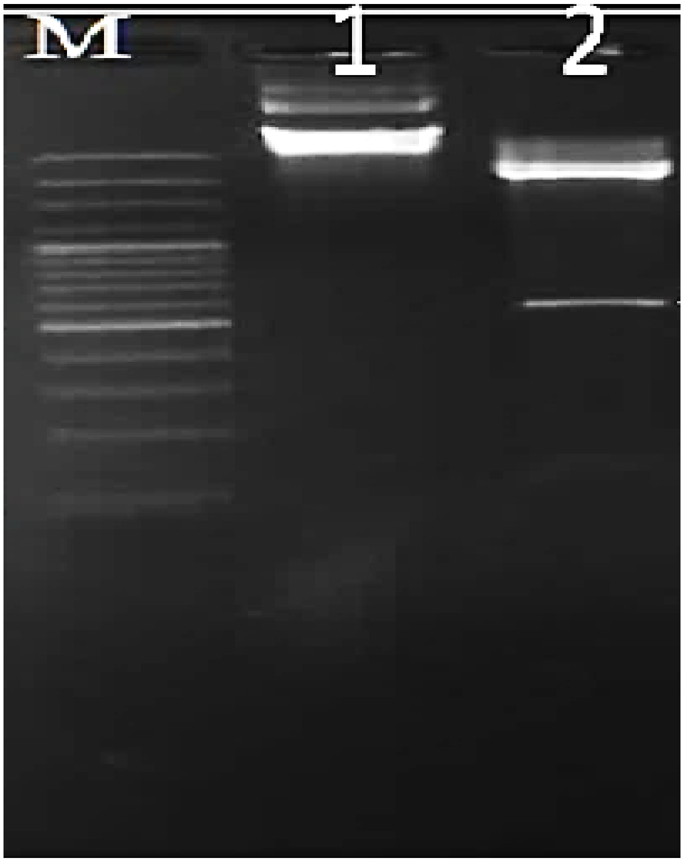

Figure S5. The recombinant antifreeze II gene. Lane 1: Plasmid DNA of the PUC57 recombinant vector contains the insert of the antifreeze gene. Lane 2: The released insert using *Eco*RI and *Eco*RV restriction enzymes.

Supplementary File (1)

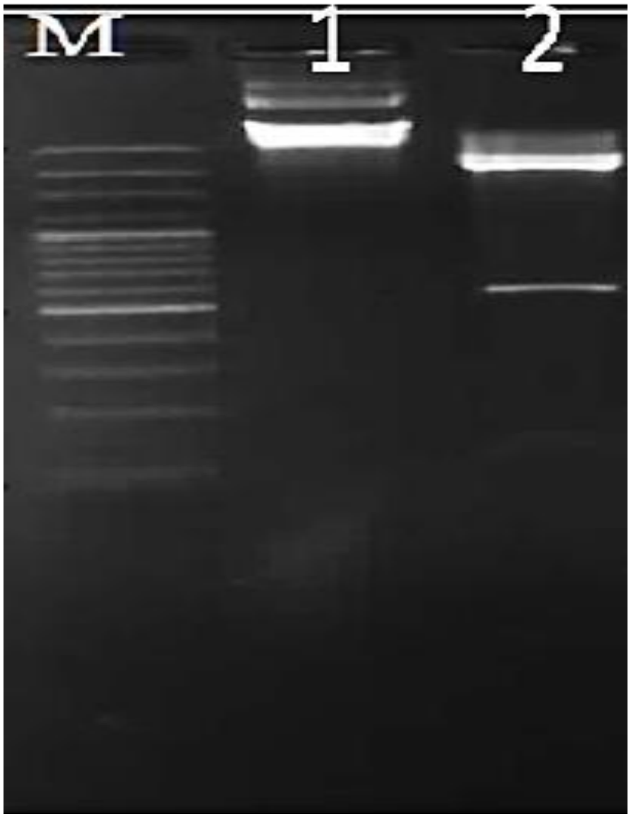

Figure S6. Plasmid DNA of the cloned antifreeze gene in TOPO TA cloning vector. Lanes; M: 5Kbp DNA ladder, Lane1: TOPO TA cloning Vector contains the antifreeze gene, Lane 2: Double digestion of the recombinant DNA plasmid.

Supplementary File (1)

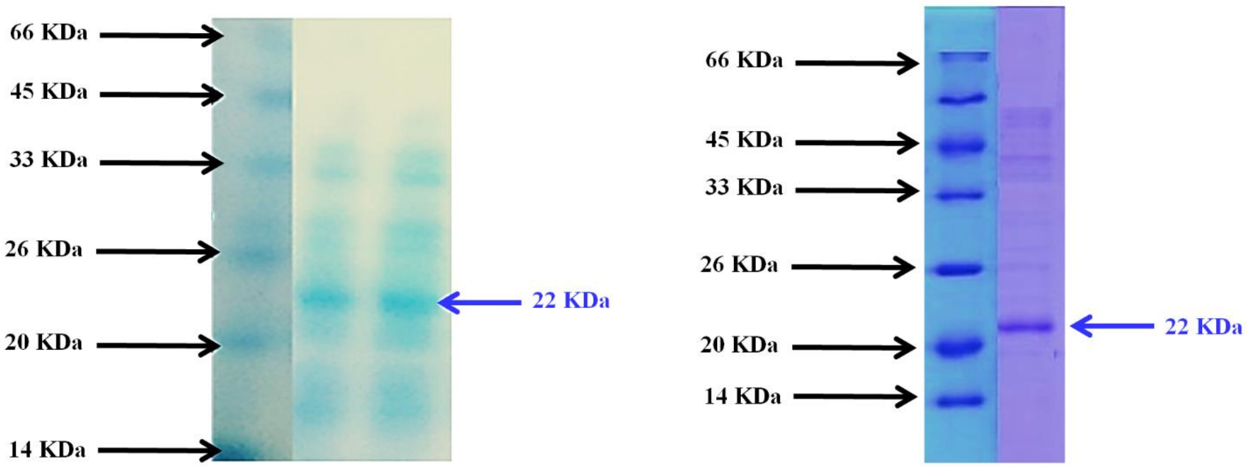

Figure S7. The recombinant protein of the antifreeze gene, Lane M: Low range protein Marker, Lane FR: partial purified recombinant protein. Note: The full-length membranes and resolution for this figure can not be provided due to the modifications that have been done to the original figures.
